# Parkinson’s disease physiopathology—beyond the *α*-synuclein aggregation: a narrative review

**DOI:** 10.3389/fnagi.2025.1664269

**Published:** 2025-12-12

**Authors:** Victor Fellipe Bispo Macedo, Vanessa Karine Bispo Macedo, Jorge Artur Peçanha de Miranda Coelho, Alana Madeiro de Melo Barboza

**Affiliations:** 1 Department of Neurology, Santa Casa de Misericórdia de Maceió, Maceió, Brazil; 2 Medical School, Federal University of Alagoas (UFAL), Maceió, Brazil; 3 Medical School, University Center of Maceió, Maceió, Brazil; 4 São Paulo University (USP), São Paulo, Brazil

**Keywords:** dysbiosis, neuroinflammation, lipid metabolism, glymphatic system, oxidative stress, pathology

## Abstract

**Background:**

Parkinson’s disease (PD) is traditionally defined by dopaminergic degeneration and *α*-synuclein aggregation. However, mounting evidence supports a multifactorial and systemic pathophysiology that extends beyond the central nervous system. This narrative review explores the interconnected mechanisms underlying sporadic PD, including environmental exposures, gut dysbiosis, *α*-synuclein pathology and propagation, systemic and neuroinflammation, metabolic dysfunctions (notably insulin and lipid metabolism), sleep disturbances, glymphatic impairment, and proteostatic failure.

**Results:**

The review highlights how *α*-synuclein pathology can originate peripherally, particularly in the enteric nervous system, and propagate to the brain via neuronal or hematogenous routes. It also examines the synergistic roles of systemic inflammation, immune dysregulation, mitochondrial dysfunction, and impaired protein clearance in promoting neurodegeneration.

**Conclusion:**

Collectively, these findings support a reconceptualization of PD as a systemic neurodegenerative disorder involving complex crosstalk between peripheral and central pathways. Understanding these multifaceted interactions opens new avenues for early diagnosis, biomarker discovery, and disease-modifying therapeutic strategies targeting the gut-brain axis, metabolic homeostasis, and proteostasis.

## 1 Introduction

Parkinson’s disease (PD) is the second most common neurodegenerative disease—surpassed only by Alzheimer’s disease (Dimoula et al., 2025; Violetta et al., 2024)—and the fastest-growing neurodegenerative condition in terms of incidence (Schmidt et al., 2023; Zhang et al., 2024). Traditionally, it has been classified as a primarily dopaminergic disorder due to the accumulation of aggregated *α*-synuclein (Costa et al., 2022). Approximately 90% of Parkinson’s disease cases are considered sporadic (sPD), resulting from complex interactions between environmental factors and genetic susceptibility (Schmidt et al., 2023).

However, recent evidence suggests that PD is now understood as a multifactorial and systemic disease (Costa et al., 2022). It goes beyond mere dopaminergic depletion in the nigrostriatal pathway and encompasses a wide range of mechanisms, including immunometabolic dysfunctions, alterations in the gut-brain axis, pathological propagation of *α*-synuclein, glymphatic system impairment, and disturbances in glucose and lipid metabolism (Dimoula et al., 2025; Zhang et al., 2024).

This review aims to highlight the main pathophysiological mechanisms underlying sporadic PD and how they interconnect to drive the progressive neurodegenerative process.

## 2 Environmental risk factors and Parkinson’s disease

Numerous environmental agents have been implicated in the etiopathogenesis of PD, with pesticides being the most strongly associated (Brolin et al., 2024; Ball et al., 2019; Dick et al., 2007). These substances, whose use has increased by 50% over the past three decades, are widely employed in agriculture, and exposure through inhalation, ingestion, or skin contact poses a significant health risk (Brolin et al., 2024; Ball et al., 2019; Bianco et al., 2025).

In general, these substances can cross the blood–brain barrier (BBB) and exert selective toxicity on dopaminergic neurons (Brolin et al., 2024; Ball et al., 2019; Elwan et al., 2006; Xiong et al., 2015; Paul et al., 2023). They promote the formation of reactive oxygen species (ROS), leading to both cytosolic and mitochondrial oxidative stress, microtubule-mediated axonal transport dysfunction, neuroinflammation, apoptosis, and the direct induction of *α*-synuclein aggregation and fibril formation (Brolin et al., 2024; Ball et al., 2019; Elwan et al., 2006; Xiong et al., 2015; Paul et al., 2023).

Other important environmental risk factors include organic solvents such as trichloroethylene—which can also cross the BBB and induce mitochondrial dysfunction in neurons, demonstrated in animal models (Brolin et al., 2024; Ball et al., 2019; Liu et al., 2010; De Miranda et al., 2021)—and air pollution, which is capable of triggering oxidative stress and inflammation initially in the lungs and nasal passages, but also systemically through the absorption of ultrafine particles into the bloodstream (Brolin et al., 2024; Ball et al., 2019; Krzyzanowski et al., 2024).

## 3 *α*-Synuclein and its pathology

*α*-Synuclein is a presynaptic protein highly abundant in the central nervous system (CNS) under physiological conditions (Dimoula et al., 2025). It is involved in synaptic neurotransmission, nucleo-cytoplasmic transport, and DNA damage repair (Burré et al., 2024). It is also present in peripheral tissues—particularly in blood cells—and is capable of crossing the BBB bidirectionally, both in its free form and enclosed within extracellular vesicles (Dimoula et al., 2025).

The exact mechanism by which *α*-synuclein aggregates remains under debate (Burré et al., 2024). However, it is known to involve protein misfolding, abnormal phosphorylation (particularly at serine 129), and oxidative modifications (Parra-Rivas et al., 2022; Ghanem et al., 2022; Andrés et al., 2016; Calabresi et al., 2023), resulting in oligomerization, fibril formation, and the development of proteinaceous inclusions (Burré et al., 2024).

This pathological process tends to occur predominantly in neurons with high energy demands and low myelination (Calabresi et al., 2023; Bartl et al., 2022). Several factors appear to contribute to this process, including increased *α*-synuclein synthesis, alterations in the intracellular environment—such as changes in pH, temperature, energy availability, ROS generation, and elevated intracellular calcium—and structural modifications of the protein that favor the formation of *β*-sheet-rich conformations (Burré et al., 2024).

Although it was traditionally believed that Lewy bodies and Lewy neurites were composed solely of aggregated *α*-synuclein, it is now recognized that these structures also contain other proteins, organelles, and cellular components (e.g., chaperones, membrane proteins, lipids and cytoskeletal elements) (Burré et al., 2024). As such, they disrupt several vital neuronal functions—including energy production, axonal transport, and cellular metabolism—ultimately leading to functional deficits, cellular stress, and apoptosis (Burré et al., 2024).

The neurons most vulnerable to this pathophysiological process appear to be those with high metabolic demands—particularly those with intense neurotransmitter release and reuptake activity—and low myelination (Calabresi et al., 2023; Bartl et al., 2022), such as the dopaminergic neurons of the substantia nigra (Ball et al., 2019).

Nevertheless, the mere accumulation of *α*-synuclein does not necessarily lead to neurodegeneration (Burré et al., 2024). Its buildup may be involved in distinct neurodegenerative mechanisms and, in parallel, may also be capable of triggering neuroprotective or compensatory responses (Burré et al., 2024).

## 4 Anatomical origin of Parkinson’s disease

Braak’s hypothesis, although not universally accepted, proposes that the pathophysiological process of PD may begin in peripheral structures—such as the myenteric plexus of the gastrointestinal tract and the olfactory bulb—and subsequently progress to the CNS (Braak et al., 2004; Ines et al., 2024). This would account for the two main premotor symptoms of PD: constipation and anosmia, respectively (Ielo et al., 2024; Jacopo et al., 2024).

Furthermore, the dissemination of *α*-synuclein along these two distinct pathways may help explain the variability in motor symptom progression observed among PD patients (Knudsen et al., 2021; Boertien et al., 2022). The hypothesis is that when dissemination occurs via the olfactory pathway, nigral involvement tends to be ipsilateral, which is more often associated with asymmetric onset of motor symptoms (Ielo et al., 2024). In contrast, when it occurs through the vagal pathway, involvement is typically bilateral due to early engagement of the bilateral dorsal motor nucleus of the vagus and, subsequently, both substantia nigra, leading to a greater tendency for symmetric motor symptoms (Ielo et al., 2024).

## 5 Gut dysbiosis and the local inflammatory process

The Westernization of the diet, rampant use of antibiotics, and exposure to pesticides have altered both the gut microbiota and mycobiota (Veronese et al., 2024; Pal et al., 2024). This microenvironment is fundamental for immune modulation and maintaining the integrity of the intestinal epithelial barrier (Barbara et al., 2021).

PD patients exhibit a reduction in taxon–taxon interactions, an increase in pro-inflammatory bacterial populations—such as Proteobacteria and Enterobacteriaceae (Hu et al., 2024)—and a decrease in short-chain fatty acid (SCFA)-producing bacteria, such as *Faecalibacterium prausnitzii* and Roseburia (Bai et al., 2024). These changes result in a reduced capacity to metabolize certain carbohydrates—particularly hexuronates, whose metabolic pathways involve the degradation of other compounds like glucuronate, which supports liver detoxification processes including clearance of toxins such as p-cresol and pesticides (Bai et al., 2024). In addition, there is a decrease in fatty acid degradation, reduced purine recycling—which is essential for maintaining adequate ADP and ATP production—and an increase in anaerobic metabolism (Metcalfe-Roach et al., 2024).

Such dysbiosis leads to alterations in gut metabolite production patterns (Zhao et al., 2024), which in turn increase intestinal permeability (“leaky gut”) by disrupting the tight junctions of enterocytes (Ines et al., 2024; Pal et al., 2024; Brown et al., 2023). An important metabolite involved in this process is lipopolysaccharide-binding protein (LBP) (Zhao et al., 2024).

One hypothesis is that the dysbiosis induces a shift in the pattern of bacterial endotoxin production (Brown et al., 2023). These endotoxins—mainly composed of lipopolysaccharides (LPS) (Brown et al., 2023)—are continuously released into the host and may exert pro- or anti-inflammatory effects, depending on the bacterial source (Brown et al., 2023).

While only a portion of these endotoxins normally cross the gut barrier and are cleared by the liver, increased intestinal permeability allows the translocation of pro-inflammatory endotoxins from the gut lumen into the intestinal wall (Brown et al., 2023). This would induces a localized inflammatory response (Ines et al., 2024; Jacopo et al., 2024; Brown et al., 2023) and further exacerbates intestinal permeability (Brown et al., 2023). Moreover, these endotoxins would be capable of directly inducing *α*-synuclein aggregation locally by promoting its expression and phosphorylation (Brown et al., 2023).

## 6 *α*-Synuclein transmission

After the emergence of pathological *α*-synuclein in the gastrointestinal tract, it can be transmitted to the CNS through two main pathways. The most classical route occurs via retrograde neuronal transport—demonstrated in both *in vivo* and *in vitro* studies, although this mechanism remain controversial—in a prion-like fashion through the vagus nerve, forming part of the gut-brain axis (Burré et al., 2024; Ines et al., 2024; Scholz and Yacoubian, 2025). Studies in prion-protein have shown that the specific kinetic mechanism of this process depend on the chemical and spatial properties of this proteins (Burré et al., 2024).

However, it is also known that a portion of this pathological *α*-synuclein can be transmitted via the hematogenous pathway, primarily through extracellular vesicles, demonstrated in animal models and patients with PD (Scholz and Yacoubian, 2025; Streubel-Gallasch and Seibler, 2023). These vesicles, composed of phospholipid bilayers, can be released by virtually all cell types and carry nuclear, lipid, and protein components from their cell of origin (Streubel-Gallasch and Seibler, 2023). Their primary function is intercellular communication, and they are capable of crossing the BBB (Streubel-Gallasch and Seibler, 2023).

## 7 Systemic inflammatory process

All these pathological processes induce a reactive inflammatory response (Calabresi et al., 2023; Jacopo et al., 2024; Pal et al., 2024; Brown et al., 2023). Initially, this response is beneficial, but when chronically sustained, it leads to local and systemic deleterious effects (Zhang et al., 2024; Jacopo et al., 2024; Pal et al., 2024; Brown et al., 2023).

Among the mechanisms involved in this pro-inflammatory state in PD patients, increased activation of toll-like receptor 4 (TLR4) and monocytes can be highlighted (Zhang et al., 2024; Brown et al., 2023)—characterized by enhanced synthesis of pro-inflammatory cytokines and chemokines (particularly TNF-*α*, IL-6, IL-8, IL-10) (Zhang et al., 2024; Brown et al., 2023)—along with increased production of neutrophils and natural killer cells, and decreased lymphocyte production (Zhang et al., 2024). There is also an increase in the pro-inflammatory Th1 and Th17 subpopulations and a decrease in the anti-inflammatory Th2 subpopulation (Muñoz-Delgado et al., 2023).

Additionally, there is greater activation of complement system receptors and reduced production of antioxidant and anti-inflammatory substances such as HDL (Zhang et al., 2024). Consequently, this pro-inflammatory state results in decreased BBB integrity and increased permeability, mainly due to the reduction of molecules such as DSG3 and SPON1 (Bartl et al., 2022), contributing to the process of neuroinflammation, as demonstrated *in vivo* study by Bartl et al. (2022).

These abnormalities can be assessed in the laboratory using ratios of certain markers, such as neutrophil-to-lymphocyte, neutrophil-to-HDL, and lymphocyte-to-monocyte ratios (Zhang et al., 2024; Muñoz-Delgado et al., 2023). PD patients present a lower lymphocyte-to-monocyte ratio and higher neutrophil-to-lymphocyte and neutrophil-to-HDL ratios (Zhang et al., 2024).

## 8 Neuroinflammation

This increased permeability of the BBB facilitates the infiltration into the CNS of endotoxins, toxic metabolites, pathological *α*-synuclein, as well as pro-inflammatory cells and molecules, initiating a process of neuroinflammation (Bartl et al., 2022; Jacopo et al., 2024; Brown et al., 2023; Muñoz-Delgado et al., 2023; Antelmi et al., 2024).

It is believed that the infiltration of these pro-inflammatory factors activates cerebral TLR4, which in turn activates microglia (Veronese et al., 2024; Brown et al., 2023). This process is potentiated by the combined action of leukotrienes on pro-inflammatory molecules such as GPR17, which are highly expressed in oligodendrocyte precursors in the CNS (Wallin et al., 2024).

Glial activation induces the production and release of pro-inflammatory cytokines—such as TNF-*α*, IL-1β, and IL-2 (Bartl et al., 2022; González-May et al., 2023)—generates oxidative stress, and activates the nitric oxide metabolic pathway, increasing the synthesis of reactive oxygen and nitrogen species via induction of nitric oxide synthase (Brown et al., 2023). It also promotes the expression of pro-inflammatory and pro-apoptotic proteins such as CCL25 and CASP8, respectively, further increasing BBB permeability, as demonstrated in human subjects with PD (Wallin et al., 2024).

As with systemic inflammation (Zhang et al., 2024; Jacopo et al., 2024; Pal et al., 2024; Brown et al., 2023), neuroinflammation begins as a physiological defense mechanism; however, persistent glial activation and excessive release of pro-inflammatory cytokines (Bartl et al., 2022; González-May et al., 2023; Al-Abdulrasul et al., 2024) ultimately could lead to the direct induction of *α*-synuclein aggregation and accumulation, neuronal apoptosis, and phagocytosis (Brown et al., 2023). Thus, neuroinflammation could be a crucial aspect in the induction of neurodegeneration, particularly in the substantia nigra, leading to progressive dopaminergic loss (Brown et al., 2023; Muñoz-Delgado et al., 2023). Three important mechanisms contribute to this neuroinflammatory process: insulin resistance (Tang et al., 2024), sleep disturbances (Antelmi et al., 2024) and alterations in lipid metabolism (Song et al., 2025).

## 9 Insulin resistance and Parkinson’s disease

It is widely recognized in the literature that there is an increased risk of PD in patients with type 2 diabetes mellitus (T2DM) (Veronese et al., 2024; Tang et al., 2024). Both diseases share several pathogenic mechanisms, including interactions between environmental risk factors and genetic susceptibility, the formation of toxic protein aggregates (such as islet amyloid polypeptide in type 2 diabetes and *α*-synuclein in PD), insulin resistance, mitochondrial dysfunction, and neuroinflammation (Tang et al., 2024; Zagare et al., 2024; Mukherjee et al., 2015).

Research with midbrain organoids demonstrated that among the various physiological functions of insulin in the CNS, not only the regulation of energy metabolism stands out, but also synaptic plasticity, mitochondrial function, and neuronal survival (Zagare et al., 2024). An important potential gene involved in part of the insulin regulatory process, which would be altered in PD patients, is FOXO1 (Zagare et al., 2024).

A contributor to exaggerated neuroinflammation is insulin resistance (Violetta et al., 2024; Zagare et al., 2024). In this condition, seems to have a activation of the phosphatidylinositol 3-kinase/protein kinase B (PI3K/AKT) pathway (Zagare et al., 2024) which promotes the release of free fatty acids and inflammatory cytokines, mitochondrial damage, oxidative stress, accumulation of toxic ceramide species—mainly C14 and C16—*α*-synuclein accumulation, and neuroinflammation (Violetta et al., 2024; Zagare et al., 2024).

Specifically in dopaminergic neurons, this condition would alters lipid regulatory metabolism, particularly cholesterol esters (Zagare et al., 2024) and the membrane lipid phospholipid-1-alkenyl,2-acyl phosphatidylcholine (PC-Os), which is involved in several cellular functions such as protein trafficking, endoplasmic reticulum health, and membrane fluidity (Zagare et al., 2024).

## 10 Sleep, glymphatic system, and Parkinson’s disease

Sleep disturbances represent an important risk factor for neurodegenerative diseases and are quite common in patients with PD (Antelmi et al., 2024). Among the various causes, some are secondary to dopaminergic dysfunction and other neurotransmitters (such as GABA) (Antelmi et al., 2024). The relationship between these two conditions is bidirectional and involves several mechanisms, including circadian rhythm abnormalities, dysfunction of the glymphatic system, neuroinflammation, and increased promotion of *α*-synuclein aggregation (Antelmi et al., 2024).

Studies in mouse model demonstrated that sleep is a key regulator of cellular protein synthesis homeostasis (proteostasis) (Antelmi et al., 2024). Thus, sleep disturbances tend to generate the production of physiologically more dysfunctional proteins—including *α*-synuclein—with a greater tendency to aggregate and accumulate (Antelmi et al., 2024).

Another contributing factor to this relationship stems from dysfunction of the glymphatic system (Yao et al., 2024). This system is composed of astrocytic endfeet rich in aquaporin-4 (AQP4) receptors, located in the perivascular space, and plays a role in the clearance of toxic brain proteins, both those produced by neuronal metabolism and pathological ones (Zagare et al., 2024). Its activity varies throughout the day, acting mainly during the N3 phase of sleep (Veronese et al., 2024).

Pathological proteins, such as aggregated *α*-synuclein, are predominantly cleared from the brain parenchyma via the glymphatic system. Cerebrospinal fluid (CSF) enters the periarterial space, traverses the astrocytic endfeet expressing AQP4, and mixes with the interstitial fluid, generating a convective flow that “washes” these solutes toward the perivenous space. The cleared *α*-synuclein then returns to the CSF and subsequently reaches the peripheral circulation mainly through the cervical lymph nodes, and to a lesser extent via the dural venous sinus (Boland et al., 2018; Yamada and Takeshi, 2024).

Thus, sleep disturbances could create a state with increased susceptibility to neuroinflammation and *α*-synuclein accumulation, contributing to the loss of various neuronal populations, such as orexin-producing neurons in the hypothalamus (Antelmi et al., 2024). Orexin plays an important role in circadian cycle regulation and sleep (De Luca et al., 2022) and has neuroprotective functions (Antelmi et al., 2024).

## 11 Lipid metabolism and Parkinson’s disease

One of the contributing mechanisms to this central inflammation process is also the dysregulation of lipid pathways—especially mitochondrial fatty acid metabolism (Song et al., 2025)—particularly involving two important sphingolipids, ceramides and sphingomyelins, as demonstrated in PD brain samples (Yang et al., 2024). Sphingolipids are present mostly in protein-rich areas of membranes in which *α*-synuclein is also located (Erskine et al., 2025). Changes in balance sphingolipid species may alter *α*-synuclein solubility and induce alfa-synucledin aggregation (Erskine et al., 2025).

Thus, metabolic dysfunction of these substances could directly trigger *α*-synuclein aggregation, which in turn hyperactivates monocytes and initiates a central inflammatory response (Zhang et al., 2024), as well as induce mitochondrial and endoplasmic reticulum dysfunction, promoting the formation of ROS (Song et al., 2025).

Sphingolipids participate in synaptic dopaminergic activity in the striatum, especially in the process of dopamine release and reuptake through modulation of dopamine transporter (DAT) function (Yang et al., 2024; Won et al., 2018). Ceramides influence dopaminergic release by modulating intracellular calcium levels and are also involved in oxidative stress mechanisms and neuronal death (Yang et al., 2024; Won et al., 2018). Sphingomyelin, in turn, is associated with a faster decline of striatal DAT, participating in dopaminergic recapture (Yang et al., 2024).

## 12 Proteostasis disturbance: ubiquitin-proteasome and autophagic-lysosomal pathways

Proteostasis integrity is essential for neuronal homeostasis (Lottes and Cox, 2020). PD is strongly associated with the failure of the main cellular systems responsible for the degradation of misfolded or damaged proteins: the ubiquitin-proteasome system (UPS) and the autophagy-lysosomal pathway (ALP) (Sahoo et al., 2022). Both systems act coordinately in the clearance of alpha-synuclein, and their dysfunction contributes to the pathological accumulation of this protein, in addition to triggering progressive neurodegeneration (Sahoo et al., 2022).

The UPS is responsible for the degradation of soluble cytoplasmic proteins through polyubiquitination and subsequent processing by the 26S proteasome (Bard et al., 2018). Neuropathological and experimental evidence indicates a reduction of proteolytic activity of the proteasome in the brains of PD patients, particularly a decrease in chymotrypsin-like activity of the 20S proteasome (McNaught and Jenner, 2001; Bi et al., 2021). Furthermore, oligomeric alpha-synuclein itself can inhibit proteasomal function, creating a vicious cycle of proteostatic overload and failure (Lindersson et al., 2004; Galka et al., 2024). Mutations in genes such as PARK2 (Parkin) and UCHL1, which encode components of the ubiquitination system, reinforce the causal relevance of this pathway in both genetic and sporadic forms of the disease (Buneeva and Medvedev, 2024; Hattori and Mizuno, 2017).

The ALP, on the other hand, is responsible for degrading aggregated proteins, dysfunctional organelles, and long-lived proteins, and comprises three subtypes: macroautophagy, chaperone-mediated autophagy (CMA), and microautophagy (Finkbeiner, 2019). Among these, macroautophagy and CMA exhibit particularly relevant dysfunctions in PD (Dehay et al., 2013). Macroautophagy is impaired in both the formation and maturation of autophagosomes, leading to the accumulation of alpha-synuclein aggregates and damaged mitochondria, with subsequent oxidative stress (Winslow et al., 2010; Magalhães et al., 2021). CMA is directly responsible for the degradation of native alpha-synuclein via interaction with LAMP-2A (Issa et al., 2018; Xilouri et al., 2013). However, mutated or post-translationally modified forms of the protein show low affinity or block lysosomal translocation, impairing CMA efficiency (Cuervo, 2004).

Moreover, primary lysosomal dysfunctions, such as those observed in mutations of the GBA1 gene, affect the activity of the enzyme glucocerebrosidase (GCase), promoting the accumulation of its lipid substrates (glucosylceramides) and stabilizing oligomeric forms of alpha-synuclein (Navarro-Romero et al., 2022). Conversely, alpha-synuclein aggregates also reduce GCase activity, creating a deleterious feedback loop for lysosomal function (Navarro-Romero et al., 2022).

The interdependence between UPS and ALP renders the joint failure of these systems even more critical, preventing proteostatic compensation and exposing dopaminergic neurons to progressive toxicity from protein aggregates (Lottes and Cox, 2020). Therefore, proteostasis impairment represents a crucial link between early alpha-synuclein accumulation events, mitochondrial failure, and terminal mechanisms of cell death (Lottes and Cox, 2020).

## 13 Neurodegeneration

The persistent inflammatory response in the CNS leads to the accumulation of pathological alpha-synuclein, mitochondrial dysfunction, energy failure, and oxidative stress, inducing degeneration of multiple neural networks (Calabresi et al., 2023; Seifar et al., 2022). Additionally, the accumulation of alpha-synuclein also results in reduced neurogenesis (Calabresi et al., 2023; Antelmi et al., 2024).

The neuronal populations most vulnerable to these processes are those with high energy demand, such as the dopaminergic neurons of the substantia nigra, noradrenergic neurons of the locus coeruleus, and cholinergic neurons of the basal nucleus of Meynert (Calabresi et al., 2023; Sun et al., 2025; Slater et al., 2024). Dysfunction of these neurotransmitter systems produces the wide array of symptoms associated with PD (Sun et al., 2025; Slater et al., 2024).

The loss of dopaminergic neurons in the pars compacta of the substantia nigra leads to impaired motor regulation and the typical motor symptoms of parkinsonism (Yao et al., 2024). However, these neurons also participate, together with noradrenaline and acetylcholine [particularly from the Ch4 cluster (Navarro-Romero et al., 2022)], in various cognitive processes as well as in the stability of posture and gait (Sun et al., 2025; Slater et al., 2024).

## 14 Final considerations

**Figure 1:**
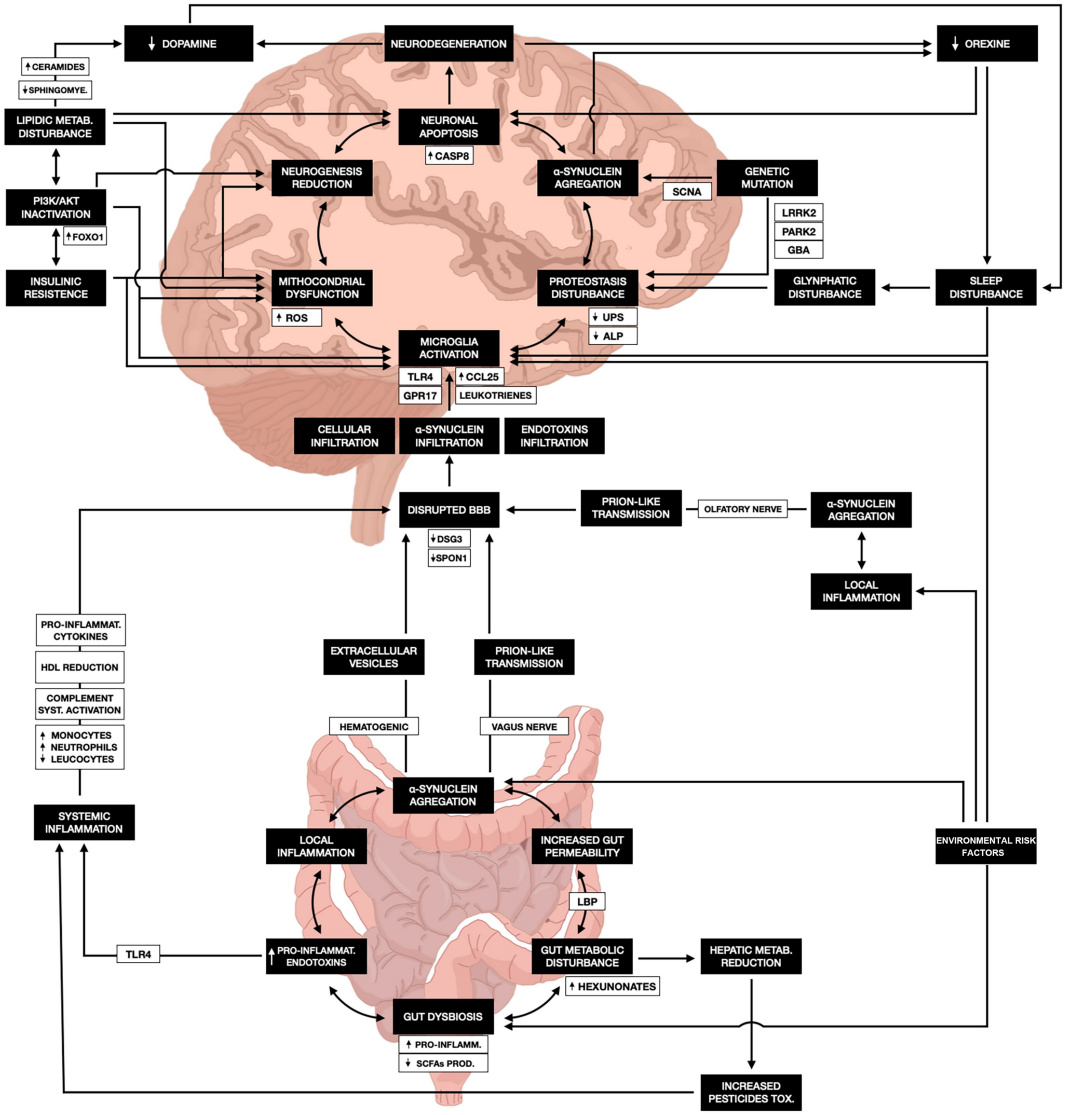
Integrated pathophysiological mechanisms linking environmental exposure, inflammation, and neurodegeneration in Parkinson’s disease. Environmental toxicants such as pesticides, solvents, and air pollutants can initiate local inflammatory responses in the intestinal or olfactory mucosa, leading to *α*-synuclein aggregation and subsequent transmission to the central nervous system via the vagus or olfactory pathways. Gut dysbiosis and increased intestinal permeability facilitate endotoxin translocation and systemic inflammation, activating TLR4-mediated microglial responses. These events promote mitochondrial dysfunction, lipid dysregulation, proteostasis impairment (UPS and ALP), and insulin resistance, resulting in oxidative stress and caspase-dependent neuronal apoptosis. Sleep disturbances and glymphatic dysfunction further exacerbate *α*-synuclein accumulation and neuroinflammation. Collectively, these mechanisms converge to drive dopaminergic degeneration and the broad spectrum of motor and non-motor symptoms characteristic of Parkinson’s disease. The black boxes represent the main pathophysiological milestones, while the connecting branches illustrate detailed mechanistic pathways involved in disease genesis. UPS, ubiquitin–proteasome system; ALP, autophagy–lysosomal pathway; TLR4, toll-like receptor 4; BBB, blood–brain barrier; ROS, reactive oxygen species.

The pathophysiological complexity of PD necessitates an integrative and transdisciplinary framework. Rather than being solely characterized as a centrally initiated dopaminergic neurodegenerative disorder, PD should be conceptualized as a systemic condition wherein peripheral and central processes—encompassing metabolic, immunological, microbiological, and proteostatic pathways—interact dynamically over time (Figure 1).

This evolving paradigm opens promising avenues for disease-modifying therapeutic strategies, including early interventions targeting the gut-brain axis, immunomodulatory approaches, restoration of energy metabolism, and enhancement of protein clearance mechanisms and glymphatic function. Furthermore, the identification of reliable peripheral biomarkers, correlating pathological alpha-synuclein species with inflammatory and metabolic indicators, holds potential to redefine early diagnosis and risk stratification.

Collectively, these insights support reclassification of PD as a multifactorial systemic neurodegenerative disorder rather than a purely synucleinopathy, highlighting the importance of integrative approaches in research and clinical management.
